# Optimization of the Surfactant Ratio in the Formation of Penta-Twinned Seeds for Precision Synthesis of Gold Nanobipyramids with Tunable Plasmon Resonances

**DOI:** 10.1021/acs.jpcc.4c08818

**Published:** 2025-02-14

**Authors:** Au Lac Nguyen, Quinn J. Griffin, Ankai Wang, Shengli Zou, Hao Jing

**Affiliations:** † Department of Chemistry and Biochemistry, George Mason University, Fairfax, Virginia 22030, United States; ‡ Department of Chemistry, University of Central Florida, Orlando, Florida 32816, United States

## Abstract

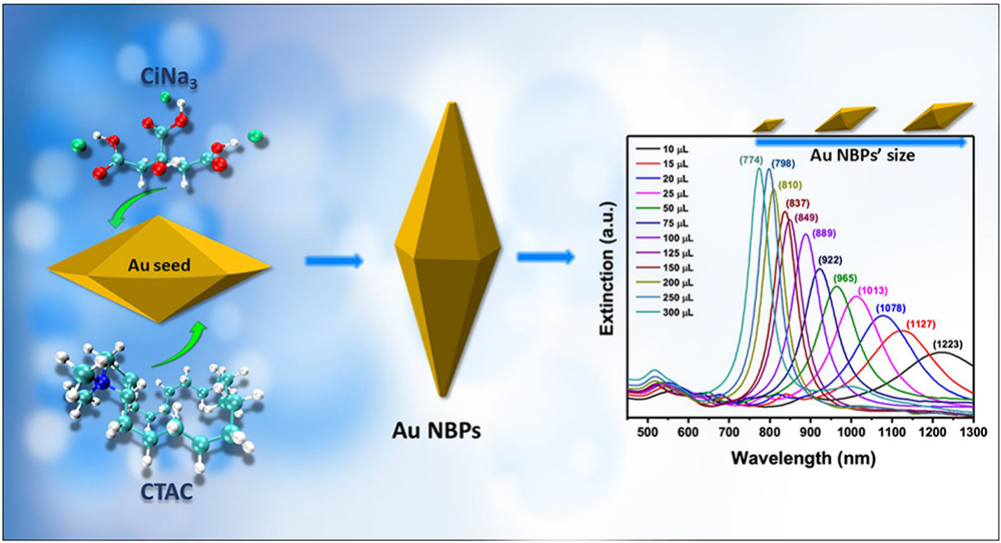

The synthesis of high-purity gold nano bipyramids (Au NBPs) with a narrow size distribution and tunable plasmon resonances is of great significance for plasmon resonance-related applications. However, the synthesis Au NBP approach involves multiple steps with many parameters that can affect the purity of the final product. In this work, we were devoted to studying the effect of the molar ratio between hexadecyltrimethylammonium chloride (CTAC) and sodium citrate tribasic dihydrate (CiNa_3_) on the seed formation stage. The results showed that the yield of Au NBP product has dramatically increased with the seed solution made from the molar ratio of CTAC:CiNa_3_ at 21:1. Furthermore, using this optimal seed, we can efficiently synthesize Au NBPs with various sizes by adjusting the concentration of the seed but keeping the rest of the parameters constant. In this study, the longitudinal localized surface plasmon resonances (LSPRs) of Au NBPs exhibit tunability beyond 450 nm across the visible and near-infrared regions from 774 to 1224 nm. We were able to successfully fine-tune the LSPRs of Au NBPs in the spectral region to become resonant with the excitation wavelengths of an 808 nm near-infrared (NIR) laser. The photothermal activities of Au NBPs were studied under 808 nm laser irradiation at ambient conditions. The present work demonstrates a paradigm for the synthesis of Au NBPs with tunable LSPRs in a precise and controllable manner, achieved by examining the surfactant ratios in the formation of penta-twinned seeds.

## 1 Introduction

Noble metal plasmonic nanoparticles have gained much more attention due to their superior physicochemical and optical properties. Surface plasmon resonance (SPR), which is the result of intense interactions of nanoparticles with light, is one of the fascinating properties of plasmonic nanoparticles. This feature leads to the particles’ spectral changes, which depend on their size and shape.^1,2^ Plasmonic nanoparticles can be employed in various potential applications in diverse fields, including biosensing,^3^ surface-enhanced Raman scattering (SERS),^4,5^ electrochemical dopamine sensors,^6,7^ drug delivery,^8^ and cancer nanotechnology.^9,10^ Furthermore, the LSPR properties can be enhanced by building blocks of hybrid heteronanostructures. This allows for increasing architectural complexity by combining two or more different plasmonic metal constituents with varying sizes, shapes, compositions, arrangements, and distributions of each component.^11,12^ Combining varying shapes of gold nanoparticles (Au NPs) with copper chalcogenide forms diverse morphologies with tunable plasmonic coupling effects.^13−15^ Notably, among the myriad of noble metal nanostructures with intriguing plasmonic properties, the anisotropic Au NBPs possessing two sharp tips have been gaining more interest owing to larger tunability in LSPRs, greater electromagnetic field enhancements, narrower line widths in the extinction spectra, and higher refractive index sensitivity, which make them more advantageous for various photonic applications, such as surface-enhanced Raman spectroscopies, refractive index change-based sensing, plasmon-mediated photocatalysis, and photothermic therapy.^16−19^

In recent years, the seed-mediated growth method has become the universal wet-chemistry approach, initially presented by Murphy and her colleagues in the 2000s,^20^ for synthesizing high-yield and monodisperse Au NBPs due to its high versatility and tunability. The typical seed-mediated growth approach includes two steps. The first step is the creation of the penta-twinned gold seeds, which function as the nucleation sites for the subsequent growth of high-yield Au NBPs. The seeds are subsequently introduced into the growing solution containing the metal precursor, surfactant, and mild reducing agent. Metal ions progressively accumulate on the seed’s surface and form particles of larger size. The surfactants stabilize and regulate the seed surface energy during this process. This can enable the metal atoms to preferentially deposit onto the designated facet, which is essential for controlling the precise size and shape of the final nanoparticles.^21−23^ In this method, it is worth pointing out that the portion of penta-twinned Au seeds plays a crucial role in the uniformity, monodispersity, and higher yield of Au NBPs with five-fold rotational symmetry about the length axis. According to early research, trisodium citrate was the only capping agent used to create low-yield penta-twinned seeds, and thermal treatment was not required, which hindered the creation of Au NBPs, and only around 30% of monodisperse bipyramids were produced.^24,25^ It was later discovered that the yield of synthesized monodisperse Au NBPs increased dramatically to approximately 60% when cetyltributylammonium bromide (CTBAB) was used in the growth solution in place of the hexadecyltrimethylammonium bromide (CTAB) stabilizing agents.^26^ However, this method can still be improved significantly by better controlling the seed crystal formation. The purity of Au NBP synthesis was notably increased using the postsynthesis purification method based on maximizing the depletion-induced self-separation. This technique significantly increases the yield of Au NBPs compared to those of the previous approaches. Nevertheless, this technique still has several drawbacks. It is usually time-consuming, uses more precursor chemicals than necessary, and may result in silver impurities on the Au NBPs surface. Because of this, the process is not feasible for large-scale manufacturing.^4,10,27^ Recently, a more straightforward method via mild thermal treatment of the seeds in the presence of binary surfactants was introduced to obtain Au NBPs with high yield and purity.^17,28^ It is essential to mention that thermal treatment also affects the morphology and purity of Au NBP synthesis by increasing the aging time to 90 min under heating around 80–85 °C. The twinning seed thermal treatment facilitated the formation of more stable penta-twinned Au seeds. In addition to thermal treatment, binary surfactants were used to improve the formation of penta-twinned Au seed in the seed synthesis step.^28−30^ Based on the well-established theories of mixed surfactants and surfactant adsorption on solid surfaces,^31^ mixing hexadecyltrimethylammonium ions (CTA^+^) and negatively charged citrate ions leads to a more complicated surfactant distribution on the surface of Au seeds. Hence, we hypothesize that the surfactant ratios in the binary mixture play a vital role in forming penta-twinned seeds, which, in turn, affects the yields of Au NBPs after implementing the available protocols for seeded growth. However, in Liz-Marzán’s work, the effect of mixed/binary surfactants on the crystallinity nature and quality of Au seeds was unclear and not well-studied.^28^

In this project, we, for the first time, systematically investigate the effect of the surfactant ratio in the binary mixture of CTAC and CiNa_3_ on the crystallinity of Au seeds for the fabrication of Au NBPs with impressive purity. We experimentally obtained the optimized molar ratio of CTAC to CiNa_3_ (21:1) to promote the high population of penta-twinned Au seeds formed in the first step of the seed-mediated approach. More importantly, using the same batch of high-quality penta-twinned Au seeds, the final bipyramidal nanostructures with varying aspect ratios from 2.4 to 3.8 by changing the volume of seed were successfully obtained, which significantly increased the reproducibility in the synthesis of monodispersed Au NBPs. The longitudinal LSPRs of Au NBPs can be finely tuned across the visible and near-infrared (NIR) spectral regions, as evidenced by an unprecedented 450 nm tunability in LSPRs in the extinction spectrum for colloidal Au NBPs. Our work is meaningful because it has been challenging, if not impossible, to decipher unambiguously the underlying universal mechanism and achieve the desired reproducibility in the two-step seed-mediated overgrowth method used for the controllable synthesis of plasmonic nanostructures due to the complexity of the numerous different chemicals involved in the growth solution. Considering the intertwining roles of surfactants, reducing agents, foreign ions or structural directing agents, and other additives in the growth solution, it is not easy to precisely obtain plasmonic nanoparticles with well-defined structures and morphologies in a highly reproducible way, especially by changing only a few parameters in the synthesis. Through our method, to synthesize monodispersed Au NBPs with tailored aspect ratios and tunable LSPRs, we can only focus on tuning the surfactant ratios in a binary mixture in the first step of the seed-mediated growth method to obtain high-quality penta-twinned Au seeds, which simplifies the synthesis of Au NBPs. We believe that this general method can be applied to the growth of other plasmonic nanostructures with increasing architectural complexity, synthesized via seed-mediated approaches. It should be noted that the insights gained from this work demonstrate a simplified paradigm for the growth of plasmonic nanoparticles in a highly controllable and reproducible manner.

## 2 Experimental Section

### 2.1 Materials

Hydrogen tetrachloroaurate (III) trihydrate(HAuCl_4_·3H_2_O), 99.99%), hexadecyltrimethylammonium chloride (CTAC, >95.0%), sodium citrate tribasic dihydrate (CiNa_3_, 99.0%), sodium borohydride (NaBH_4_, 99.99%), hexadecyltrimethylammonium bromide (CTAB, >98.0%), l-ascorbic acid (AA ≥ 99.0%), silver nitrate (AgNO_3_), hydrochloric acid (HCl), distilled ethanol (C_2_H_5_OH, >99.99%), and 4-aminothiophenol (p-NTP, grade 80.0%) were all purchased from Sigma-Aldrich. All chemicals were used as received without further purification. Ultrapure deionized water (Milli-Q, 18.2 MΩ·cm at 25 °C) was used in the experiment.

### 2.2 Synthesis of Gold Seeds

The gold seed solution was prepared by using the reduction method. HAuCl_4_ (5 mL, 0.25 mM) was quickly reduced by freshly prepared ice-cold NaBH_4_ (0.125 mL, 25 mM) in an aqueous solution containing CTAC (2.5 mL, 200 mM) and 2.375 mL CiNa_3_ with varying concentrations to obtain the ratio between CTAC: CiNa_3_ 42:1, 21:1, 14:1, 10:1, 8.4:1, and 7:1 under vigorous stirring at ambient temperature for 2 min. The color of the gold seed solution changed from faint yellow to brownish. The mixture was then placed in an 80 °C oil bath under stirring at 300 rpm. After aging for 2 h, the resultant gold seed solution changed to a translucent red color. The gold seed solution was removed from the oil bath and stored at room temperature.

### 2.3 Synthesis of Gold Nano bipyramids

It should be mentioned that the seed shape determines the most crucial step in the synthesis of high-yield Au NBPs. In this work, Au NBPs were synthesized by using seed-mediated growth processes. Briefly, the Au NBPs were formed in a solution containing 10 mL CTAB 100 mM, 0.5 mL HAuCl_4_ 10 mM, 0.1 mL AgNO_3_ 100 mM, 0.2 mL HCl 100 mM, and 0.08 mL AA 100 mM. Then, 10–300 μL of the as-prepared seed solution was quickly injected into the growth solution. The growth solution was gently stirred for 2 min and was left undisturbed in an oil bath at 30 °C for 2 h. Then, the colloid was washed 2 times at 6000 rpm for 6 min with ultrapure water. The residue was redispersed in 10 mL CTAB 5 mM and saved for further use.

### 2.4 Characterization and Instrumentation

The optical extinction spectra of the colloidal gold seed and gold nano bipyramids were recorded using a Shimazu UV-2600 spectrophotometer at an ambient temperature, equipped with quartz cuvettes of 1.7 mL volume. The size and morphological structure of the obtained Au NBPs were observed using a transmission electron microscope (TEM, JEOL JEM-1400Flash) operating at 120 kV. High-resolution TEM (HR-TEM, JEOL) was also utilized to analyze the morphological structure of the gold seed, operating at 200 kV.

### 2.5 Photothermal Evaluation of Au NBPs

The photothermal effect of Au NBPs in water with various particle sizes was determined by using an 808 nm laser. In particular, 1.5 mL of Au NBPs in ultrapure water solution containing 1 cm long with four transparent side quartz cuvettes was prepared. Then, it was irradiated for 40 min with a 2.0 W cm^–2^ continuous near-infrared laser (MDL-H-808-5W, Changchun New Industries Optoelectronics Tech Co, Ltd., China). The temperature of each sample was recorded every 5 min by utilizing an infrared (IR) thermal imaging camera (TG165-X, FLIR, Taiwan). Additionally, the sample was exposed to the 808 nm laser for 20 min. Then, it was allowed to cool for 20 min. This process was repeated three times in order to examine the suspension stability of Au NBPs.

### 2.6 Discrete Dipole Approximation Simulation

We used the discrete dipole approximation (DDA) method to model the optical spectra of Au bipyramid nanoparticles.^32^ In the DDA method, the particle is divided into N polarizable cubes. The particle, composed of these N cubes, is generated based on parameters from experimental measurements of bipyramids with C5 symmetry. The length of each cube was set to be one nanometer. The sharp tips of the bipyramids at both ends were slightly cut to mimic the synthesized nanoparticles. The dielectric constant of Au from Palik’s Handbook was used in the simulations, and the environment was simulated as water.^33^

### 2.7 Density Functional Theory Calculation

Density functional theory (DFT) was employed for theoretical simulations of Au (111), (110), and (100) surfaces as well as the adsorption of CiNa_3_ and CTAC on different Au surfaces. To simulate different Au surfaces, bulk Au cubic was first simulated and fully relaxed, and then Au (111), (110), and (100) surfaces were extracted from the relaxed bulk structure. We used k-point meshes of 3 × 3 × 1 for a 4-layer (2 × 2) Au (111) surface, 2 × 2 × 1 for a 9-layer (2 × 2) Au (110) surface, and 2 × 2 × 1 for a 5-layer (3 × 3) Au (100) surface. A 15 Å vacuum layer was applied for all Au surfaces. The Perdew–Burke–Ernzerhof functional^34^ was employed in all DFT simulations, and the pseudopotential was described by the ultrasoft method.^35^ The plane-wave basis set was expanded with a 46 and 500 Ry kinetic energy cutoff and charge density cutoff, respectively. To account for long-range interactions, the DFT-D3 method of Grimme correction^36^ was adopted in all DFT calculations. All DFT calculations were carried out using the Quantum Espresso 7.1 package,^37^ and Visualization for Electronic and Structural Analysis (VESTA) 3.5.7 software^38^ was used for result visualization. The CTAC molecule was modified to reduce computational costs by shortening the alkane tail to a propyl group attached to the nitrogen head. CiNa_3_ and the modified CTAC molecule were relaxed in a 25 × 25 × 25 Å cubic cell. The adsorption energy of capping agents on different Au surfaces was calculated using the following equation:
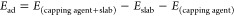


## 3 Results and Discussion

**Figure 1:**
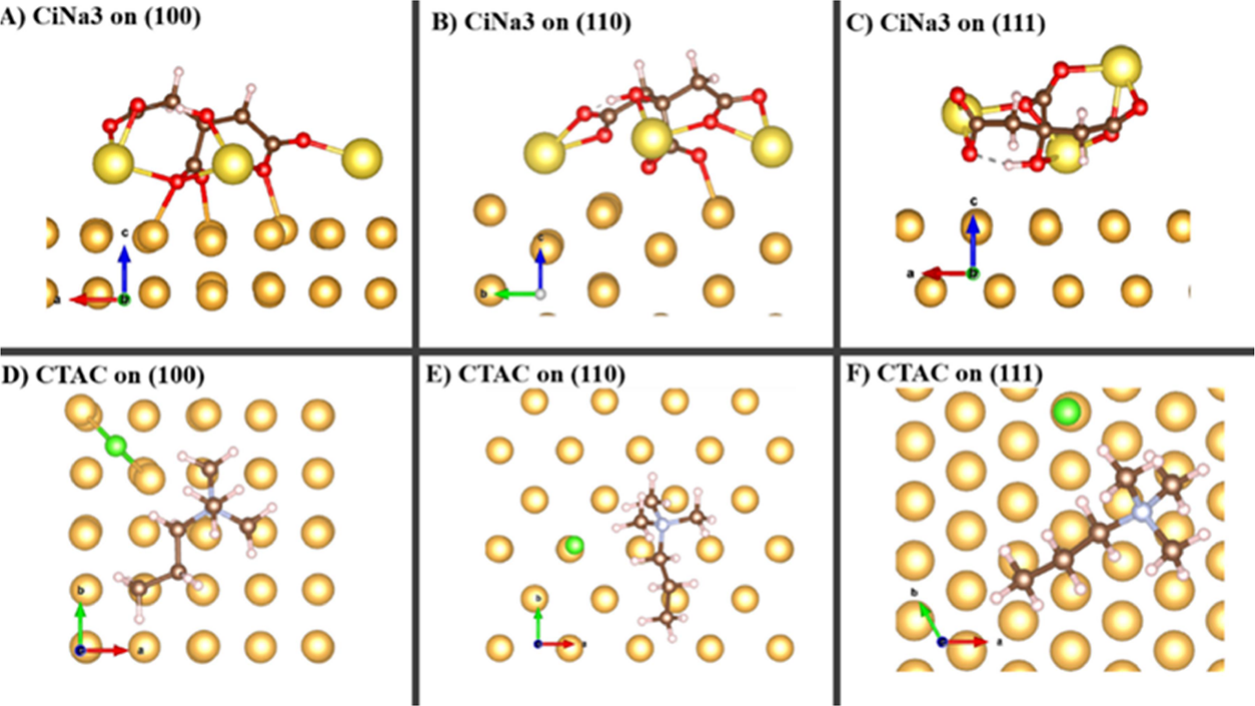
Adsorption energies of CiNa_3_ and CTAC molecules on the different Au facets calculated by the DFT method.

Figure 1 shows the adsorption energies of CiNa_3_ and CTAC molecules on different Au facets (100, 110, and 111) by using DFT calculations. The adsorption energy of CiNa_3_ on Au (111) is −36.34 kcal/mol, which is higher than that on Au (100) and (110), accounting for −64.65 and −61.49 kcal/mol, respectively (Figure 1A–C). Consequently, the higher packing densities of CiNa_3_ molecules on Au (100) and Au (110) facets further suppress the deposition of Au atoms in the formation of Au nano seeds. This, in turn, induces the growth tendency of Au atoms along the (111) direction in forming Au nano seeds. A similar trend in forming Au seeds along the (111) direction using CTAC surfactant molecules is confirmed in Figure 1D–F. CTAC molecules adsorb less favorably on the (111) direction than on the (100) and (110) directions, with the adsorption energy of −42.86 kcal/mol observed on Au (111), while Au (100) and (110) experience energies of 53.11 and −47.49 kcal/mol, respectively. This facilitates the formation of Au seeds preferably enclosed by (111) facets. These DFT results suggest that Au seeds enclosed by (111) are more energetically favored by using either CiNa_3_ or CTAC molecules.

The high yield and quality of the monodisperse Au NBPs were successfully synthesized by using the seed-mediated growth approach. Additionally, Liz-Marzán’s work^28^ demonstrated that a binary surfactant at an elevated temperature in the first step of the seed-mediated method facilitated the formation of penta-twinned Au nano seeds. To better understand the impact of the molar ratio and determine the optimal molar ratio between CTAC and CiNa_3_, we conducted a number of trials using different CTAC to CiNa_3_ molar ratios. To be more specific, we focused on improving the formation yield of penta-twinned seed by adjusting the molar ratio between CTAC and CiNa_3_ as binary surfactants under the same reaction conditions at 80 °C for 120 min. First, six different seed solutions were prepared by changing the concentration of CiNa_3_ only, while other parameters remained unchanged. Hence, the difference between these seeds is the variation of the molar ratio between CTAC and CiNa_3_ from 7:1 to 42:1, represented by 30–5 mM of CiNa_3_, respectively, while the concentration of CTAC 200 mM was kept constant.

**Figure 2:**
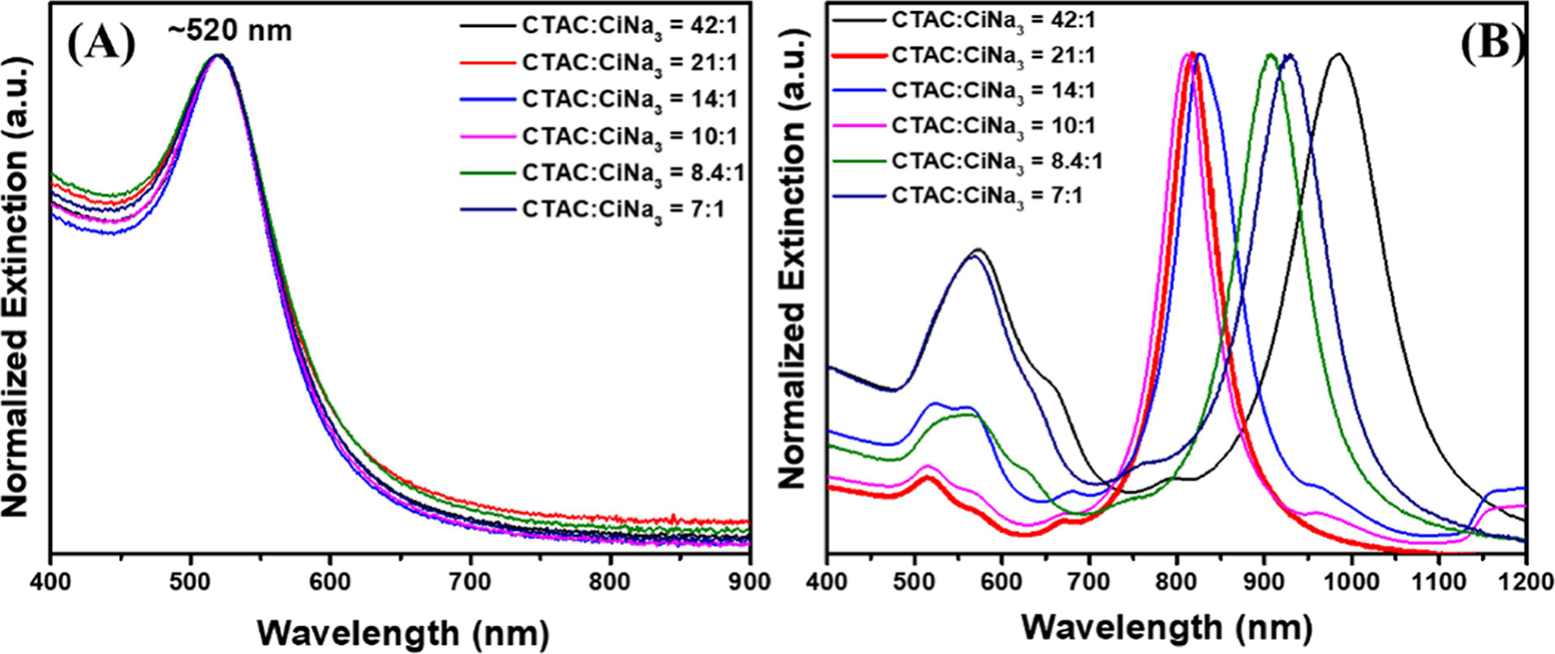
CTAC and CiNa_3_ molar ratios affect the yields of Au NBPs. (A) UV–vis spectra of seed solutions of varying CTAC:CiNa_3_ molar ratios. (B) UV–vis-NIR spectra of samples prepared using seed solution series with varying CTAC:CiNa_3_ molar ratios.

**Figure 3:**
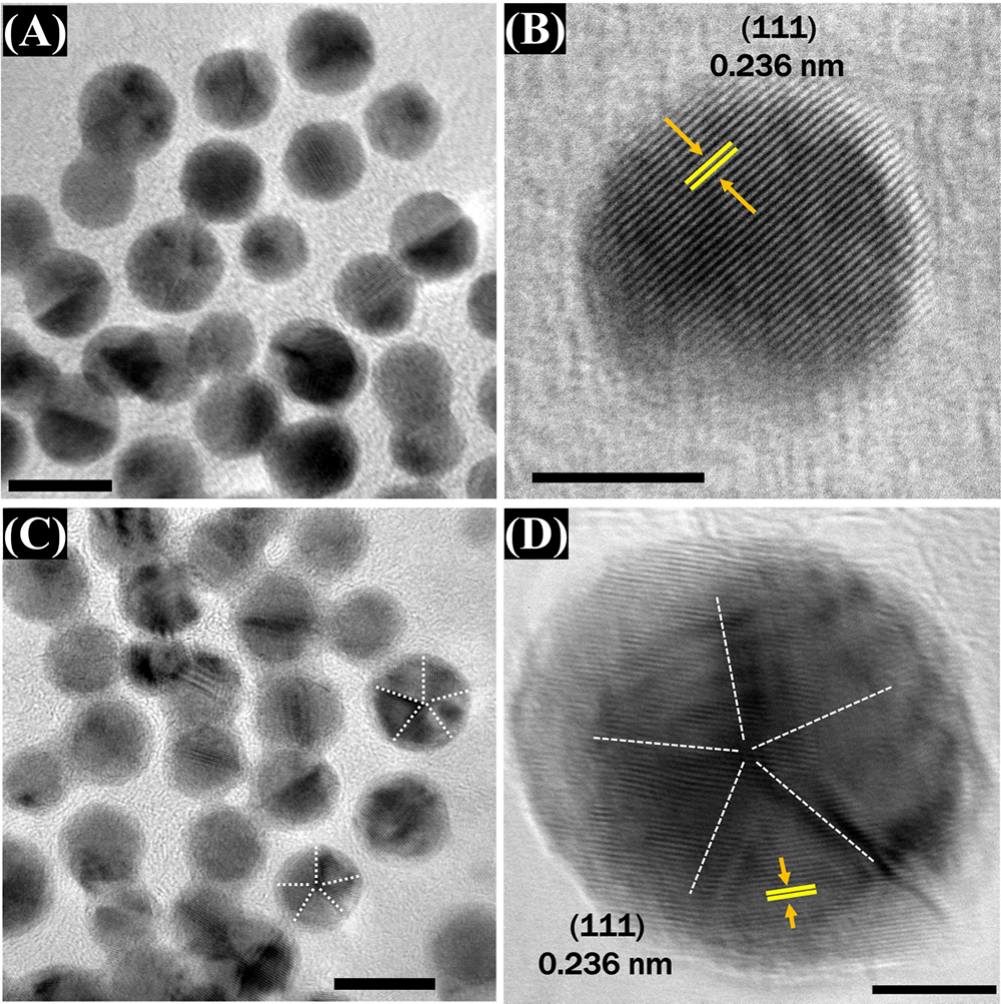
HR-TEM images of two types of gold seeds with different CTAC: CiNa_3_ molar ratios. (A,B) Single-crystal seed with a CTAC: CiNa_3_ ratio of 42:1 and (C,D) multiply twinned seed with a CTAC: CiNa_3_ ratio of 21:1. The scale bars are (A,C) 10 nm, and (B,D) 5 nm.

**Figure 4:**
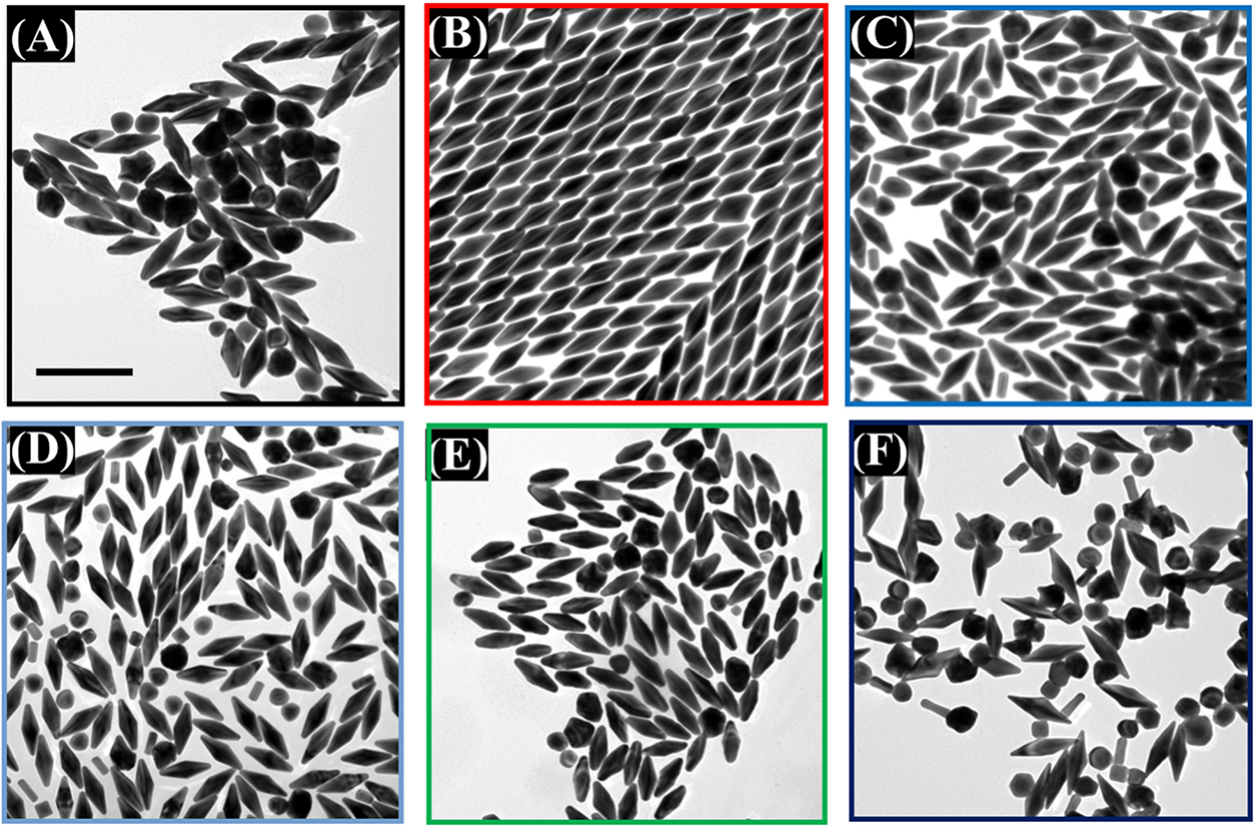
TEM images of monodisperse gold bipyramids synthesized using the seed solution with various CTAC:CiNa_3_ molar ratios: (A) 42:1; (B) 21:1; (C) 14:1; (D) 10:1; (E) 8.4:1; and (F) 7:1. The scale bar 200 nm.

Figure 2A illustrates that the absorbance and intensity peaks of the as-synthesized Au seeds were very consistent for all various CTAC: CiNa_3_ ratios, exhibiting a distinct peak centered at around 520 nm with similar single broad LSPRs in their optical extinction spectra. It is strongly confirmed that the seeds have similar sizes and homogeneous shapes.^1,6^ The monodispersed Au nano seed formation is also confirmed using HR-TEM, wherein two different Au seeds have similar sizes of around 8 nm (Figure 3A,C). Moreover, the crystallization formations of seeds obtained from different CTAC: CiNa_3_ molar ratios are also observed. Figure 3A,B demonstrates that most Au nano seed particles with a 42:1 CTAC: CiNa_3_ molar ratio exhibited a single-crystal formation. However, this seed solution has a small amount of singly twinned structure formation. Figure 4A shows that the yield of Au NBPs synthesized from this seed solution is incredibly low. Noticeably, the high polycrystallinity of penta-twinned Au nano seed was obtained with a 21:1 CTAC: CiNa_3_ molar ratio, as shown in Figure 3D. Here, citrate ions serve as etching inhibitors, surface-capping stabilizers, and reducing agents, all of which are crucial functions. They contribute to controlling the seed’s penta-twinned particle formation.^39,40^ Additionally, CTAC has two roles when absorbing onto the surface of nanocrystals: functioning as a stabilizing and surfactant and serving as a vital shape-directing agent. As a result, the growing nanocrystals are stabilized and controlled. The chloride ions in CTAC induce gold nanocrystals to anisotropic growth through selective binding to specific facets with varying surface energies.^41,42^ These surface-capping agents offer greater control of forming penta-twinned seeds. However, they will work perfectly when paired with the ideal ratio. Interestingly, the high yield of monodispersed Au NBPs increases significantly when the penta-twinned Au nano seed population is improved.^24,43^ The Au NBPs are merely formed from the penta-twinned particles.^44^

The second step of the method involves reducing the gold precursor in the presence of a silver salt, a mild reducing agent (ascorbic acid), and a surfactant (CTAB) to produce the Au NBPs from the seed solution.^45^ As mentioned in more detail in the experiment section, extinction spectra of the synthesized Au NBPs versus the same volume of varying seed solutions are shown in Figure 2B. It can be seen that two distinguishing peaks appear in the extinction spectra. The Au NBPs made from seeds at different CTAC:CiNa_3_ molar ratios exhibit similar intense and blue-shifted longitudinal LSPRs, but with various intensities of the LSPR band around 550 nm. Specifically, the samples at CTAC:CiNa_3_ (42:1; 14:1; 10:1; 8.4:1; and 7:1) molar ratios show higher intensity of the LSPR band at this position than the sample with a CTAC: CiNa_3_ molar ratio at 21:1. It has been demonstrated that a higher intensity of the extinction peak around 550 nm indicates that a higher presence of isotropic byproducts or impurities in the sample.^1,46^ According to the absorbance behavior, Au NBPs from seed solutions made with a CiNa_3_ concentration of 10 mM display the lowest longitudinal band at about 550 nm, which is indicative of a higher Au NBP yield and lower shape impurity.^47^

Furthermore, the optical features indicated above are substantially supported by the morphological characteristics of the as-prepared Au NBPs, as seen by TEM in Figure 4. The seed solution with an optimal CTAC: CiNa_3_ molar ratio of 21:1 ratio was used to produce excellent quality Au NBPs in both monodisperse and shape yields (Figures 4B and S1, SI). On the other hand, TEM images of the rest of the samples were made from different seeds with varying CTAC: CiNa_3_ ratios still remain very high shape impurities such as nano spherical, nanorod, nanocube, or nano decahedron particles in samples (Figure 4A,C–F). The synthetic yield of Au NBPs increased significantly by adjusting the binary surfactant ratios in the initial stage of the seed-mediated growth process to produce high-quality penta-twinned Au nano seeds.

**Figure 5:**
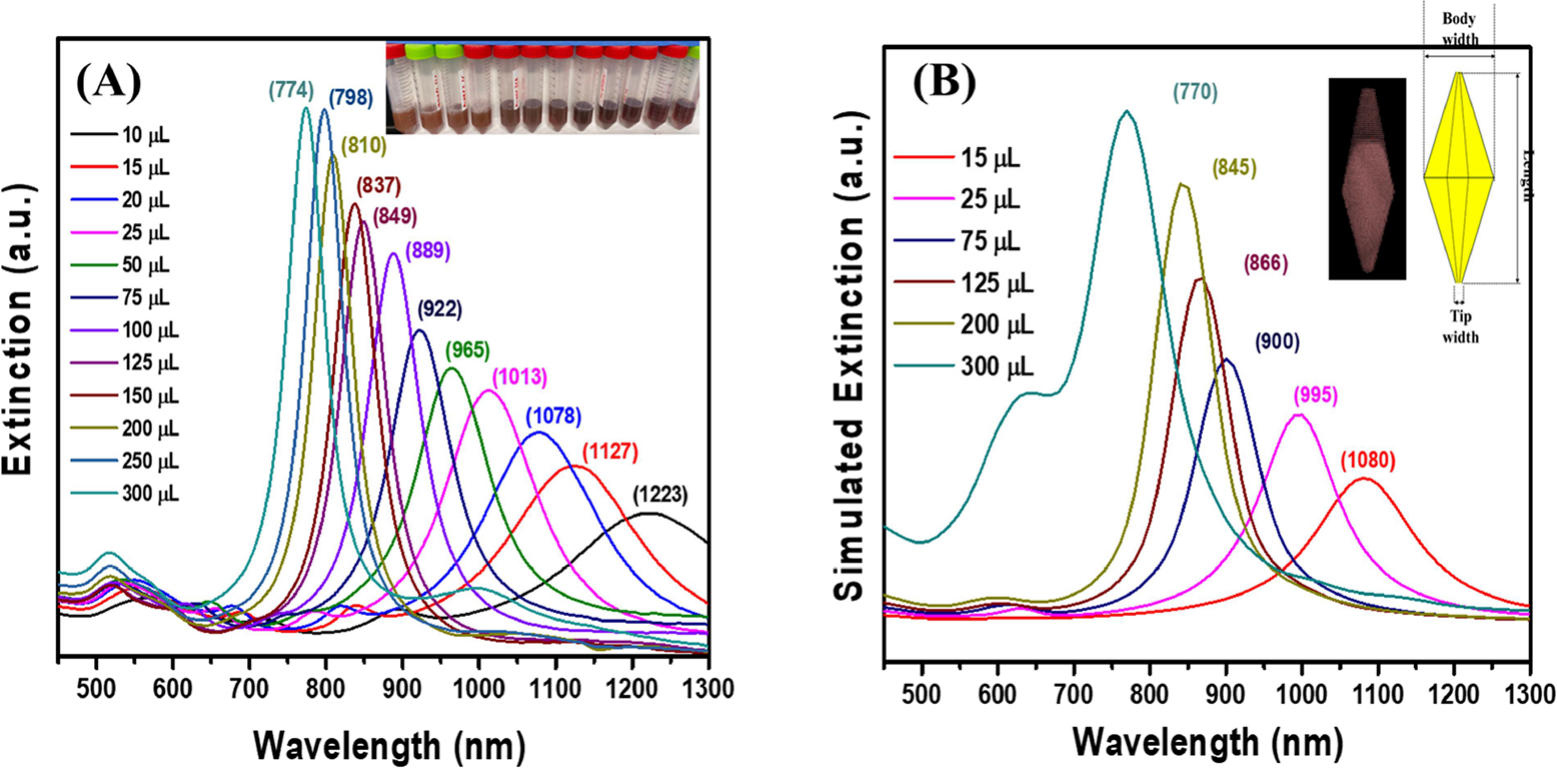
(A) UV–vis and NIR spectra of Au NBP samples synthesized using varying volumes of the optimized seed solution. (B) Simulated extinction spectra of the Au NBP samples with varying amount optimized seed solution volumes 15, 25, 75, 125, 200, and 300 μL.

Following the successful synthesis of a high yield of homogeneous Au NBPs, with a focus on the role of the seed by only adjusting the molar ratio between two capping agents CTAC:CiNa_3_ to 21:1. We subsequently synthesized anisotropic Au NBPs of various sizes by using merely different amounts of the optimal seed solution volume in order to thoroughly study the relationship between the aspect ratio and the longitudinal dipolar plasmon wavelength. The optical extinction spectra of obtained Au NBPs of various sizes show that there is a very small peak around 550 nm. This demonstrated that all Au NBPs samples obtained extremely high purity. Furthermore, the plasmon resonance of Au NBPs was readily tuned by a lower concentration of seed produced higher longitudinal LSPR band position, as seen in Figure 5A. The longitudinal LSPRs of Au NBPs exhibit tunability beyond 450 nm across the visible and near-infrared regions. Additionally, the experimental results are supported by the extinction spectra of the Au NBP samples, with shape parameters of Au NBPs from varying volumes of the optimal seed solution, computed by using DDA simulations. They exhibit excellent agreement with experimental data regarding changes in the relative intensities and the locations of the longitudinal and transversal plasmon resonances. It is crucial to note that these high-quality Au NBPs in a range of sizes were synthesized by using varying quantities of the optimal seed solution, with all other parameters remaining constant throughout a single growing stage and without a purification step. In addition to the two primary longitudinal and transverse plasmon resonance peaks, there are additional plasmon resonance peaks between the two. Notably, the additional peaks shift in the same direction as each sample’s longitudinal LSPR band. These LSPR peaks were caused by multipole plasmon resonance and were seen in high aspect ratio Au NBPs spectra. The distribution of polarized charges and the charge pairs at and close to the two tips may be explained.^16,48,49^

**Figure 6:**
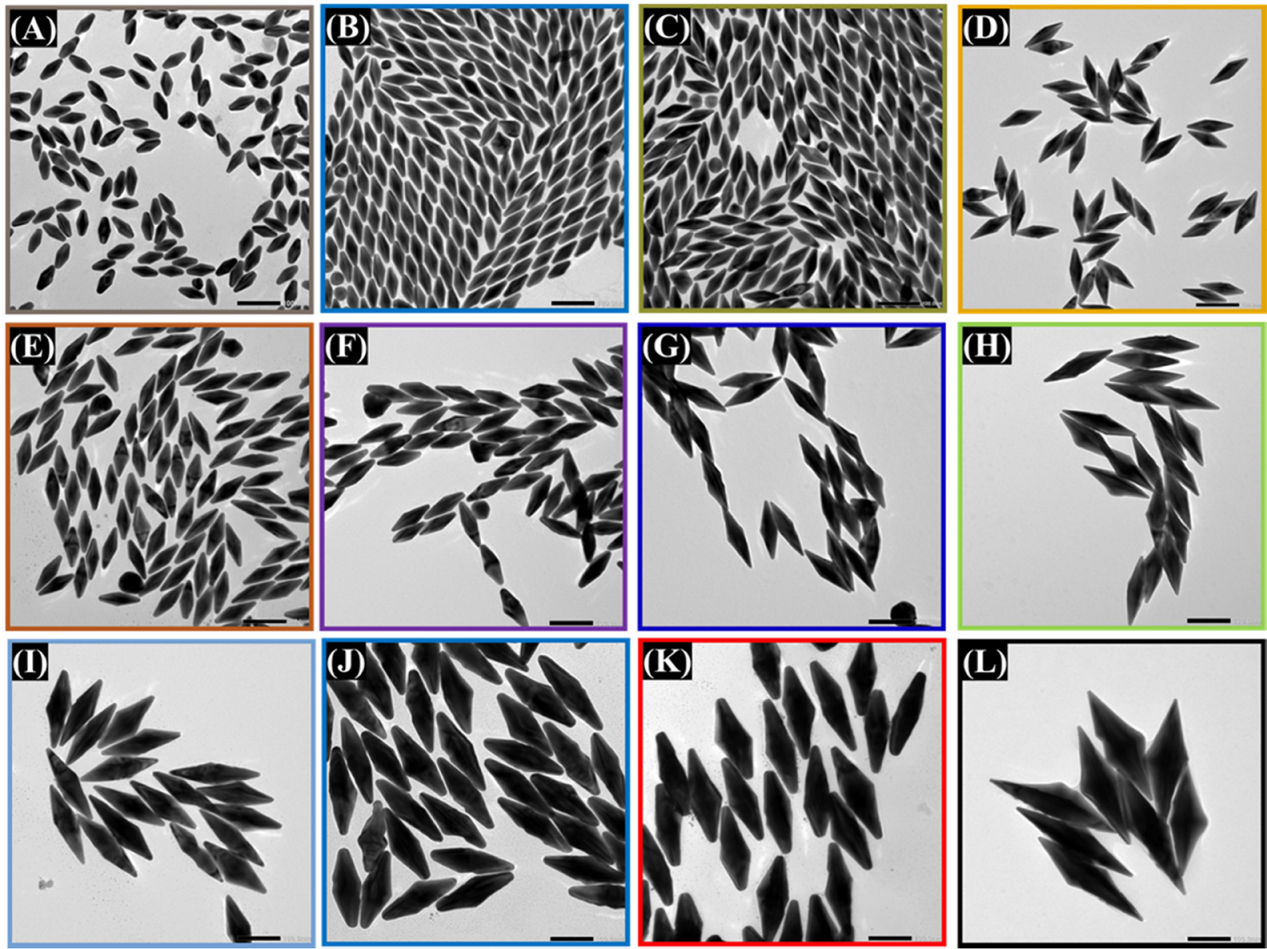
TEM images of Au NBPs of various sizes were synthesized by using various volumes of optimized seed solutions. (A) 300 μL, (B) 250 μL, (C) 200 μL, (D) 150 μL, (E) 125 μL, (F) 100 μL, (G) 75 μL, (H) 50 μL, (I) 25 μL, (J) 20 μL, (K) 15 μL, and (L) 10 μL. The scale bar is 100 nm.

**Figure 7:**
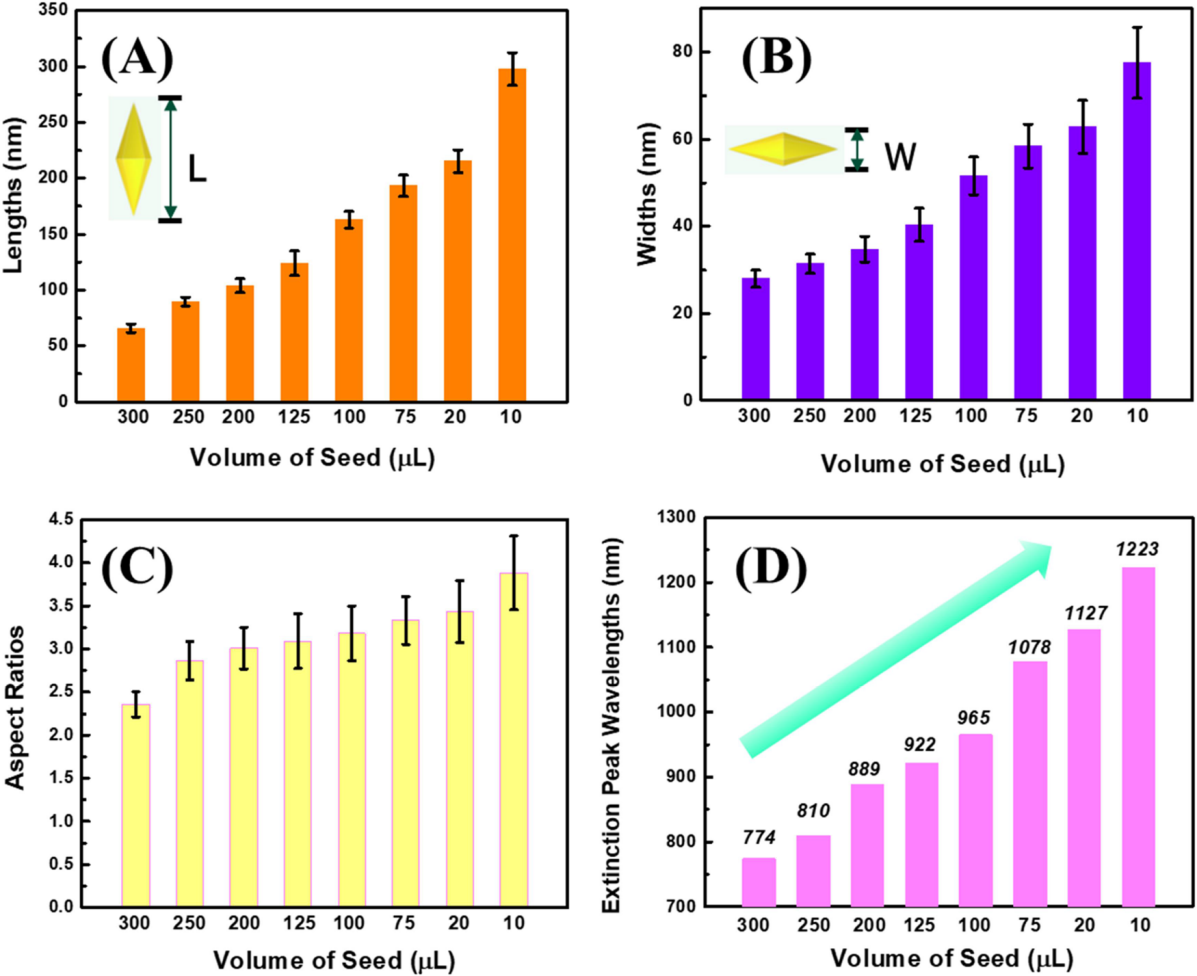
Size distribution of Au NBPs of samples prepared using different amounts of the optimized seed solution. (A) Length distribution, (B) width distribution, (C) aspect ratios, and (D) extinction trend.

Remarkably, as can be seen in Figure 6, uniform Au NBPs with various sizes can be obtained by changing the amount of optimal seed solution. The length and width of the Au NBPs increased consistently from 65.7 ± 3.7/27.9 ± 2.9 to 297.8 ± 14.7/77.5 ± 8.2 nm with decreasing seed concentration from 300 to 10 μL respectively, as demonstrated in Figure 7A,B. When the amount of seed volume decreased, it not only increased the size of Au NBPs in both length and width but also the tips became sharper. Specifically, with the highest seed concentration used in this study (300 μL), the tips of the Au NBPs showed the largest truncation. On the other hand, in the case of the lowest amounts of optimal seed solution at 10 μL, sharp edges were observed, and the longitudinal LSPRs were clearly shifted to the NIR region at 1223 nm, as depicted in Figure 5A. The final size of the Au NBPs is greatly influenced by the quantity of seeds. The final particle size increases with a smaller number of seeds introduced to the growth solution. Because more precursor gold is deposited on the particle growth, fewer nucleation sites of gold atoms are used. Conversely, a higher seed concentration results in a greater number of seed particles; less precursor gold metal is well-distributed among them, and ultimately, the particles are smaller in size.^50,51^ In addition, all synthesized Au NBPs have highly uniform and well-defined bipyramid geometry with narrow size distribution, as depicted in the size distribution histograms (Figures S5–S7, SI). The standard deviation of most of the samples is around 5%, which indicates that the size of the Au NBPs in our research was well-controlled by simply adjusting merely the concentration of the optimal seed solution. It is essential to mention that the optimal seed was prepared by adjusting the molar ratio of binary surfactants.

As illustrated in Figure 7C, the aspect ratio (AR) of the Au NBPs could be adjusted from 2.4 to 3.8 by varying the seed injection volume. The AR increased progressively as the seed volume decreased. Specifically, when the growth solution was mixed with 300 μL of seed solution, the AR was as low as 2.4. The AR considerably increased to 3.8 when we decreased the seed solution volume to 10 μL. Furthermore, the longitudinal LSPR peaks demonstrated a consistent shift toward longer wavelengths, from the visible to the near-infrared area, as the aspect ratios increased, which has been demonstrated in previous literature.^52^ According to Figure 7D, the AR was obtained between 2.4 and 3.8, corresponding to a longitudinal resonance peak from 774 to 1224 nm with a 450 nm tunability. It is essential to highlight that the LSPR tunability of the Au NBPs in this study has applications in biomedical imaging, biosensing, and photothermal therapy at specific wavelengths.

**Figure 8:**
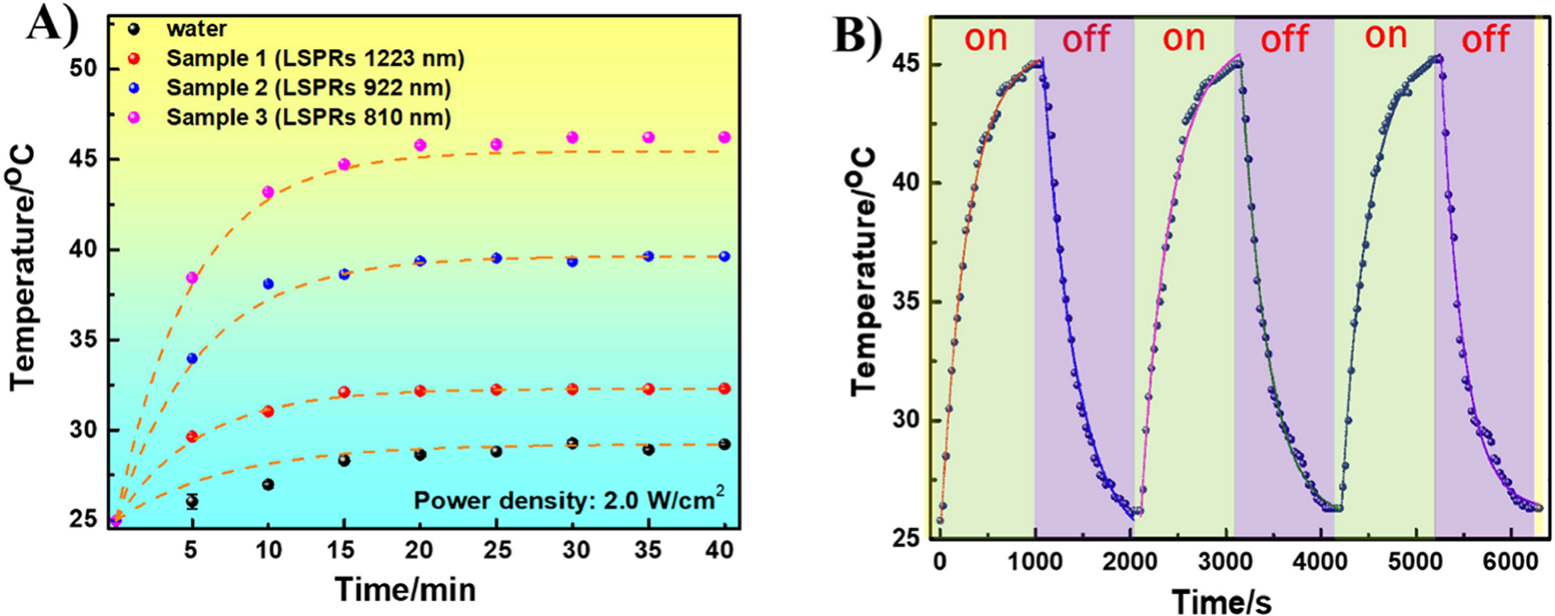
Temperature elevator curve of Au NBPs dispersed in ultrapure water prepared using 10 μL (sample 1), 75 μL (sample 2), and 200 μL (sample 3) of the optimized seed solution, and ultrapure water over 40 min under irradiation with the NIR laser at 808 nm. (B) Photothermal stability test of Au NBP sample 3 exposed to 808 nm laser irradiation for three cycles.

We then evaluate the absorption properties of the Au NBPs, which show an incredibly significant electric field amplification at both sharp tips, multiple times higher than those of gold nanorods. The higher the absorption cross section, the higher the photothermal effect.^26,53^ Due to their distinct geometry, Au NBPs provide additional advantages in photothermal therapy applications.^54,55^ We carried out four experiments with one ultrapure water sample and three Au NBP samples, which display three distinct LSPRs at 810, 922, and 1223 nm, which were synthesized using different seed volumes ranging from 200, 75, and 10 μL, respectively. These samples were continuously illuminated under irradiation with the NIR laser at 808 nm for 40 min. The temperature of each sample was carefully recorded every 5 min using an IR thermal imaging camera. Figure 8A shows that the temperature of the Au NBPs suspended in water grew progressively under laser illumination, peaking after 40 min. The temperature of Au NBP sample 3, which was prepared from 200 μL of seed solution, increased to 46.2 °C, which was much higher than the temperatures of sample 2, sample 1, and ultrapure water, accounting for 39.6, 32.0, and 29.0 °C, respectively. The localized surface plasmon resonances (LSPRs) of sample 3 at 810 nm closely match the excitation wavelength of the laser (808 nm). This resonance induces conduction electron oscillations on the surface of the Au NBPs, leading to enhanced absorption at this specific wavelength. Due to the strong interaction of the Au NBPs with light at their plasmon resonance wavelength, the absorbed energy is primarily dissipated through nonradiative relaxation processes. These processes rapidly transfer the electron energy to the particle’s metallic lattice, exciting phonons and causing thermal vibrations. The energy is then converted into heat, which increases the temperature of the Au NBPs. As a result, the localized temperature around the Au NBPs rises, and this heat is subsequently transferred to the surrounding medium.^56^

A photothermal stability test was then performed on Au NBP sample 3, which was exposed to 808 nm laser irradiation for three cycles. After 20 min of illumination under the 808 nm laser, the sample’s temperature rose to 45.2 °C. The laser source was then turned off to allow natural cooling for the same period. These results, as shown in Figure 8B, were comprehensive, with no discernible differences between the three cycles and in perfect agreement with the earlier result.

## 4 Conclusions

In conclusion, we successfully demonstrated a convenient and cost-effective method for fabricating highly uniform Au NBPs by simply adjusting the molar ratio of binary surfactants in the seed preparation step. We discovered that the optimal molar ratio between CTAC and CiNa_3_ is 21:1. The seed was prepared using the optimal ratio, forming a high purity of penta-twinned seeds. This ratio plays a crucial role in Au NBPs and significantly increases the yield of the synthesis process to facilitate studies that require high uniformity of Au NBPs. Additionally, it is essential to mention that the size of the Au NBPs can be finely and precisely adjusted by regulating the seed concentration in the growth solution. The longitudinal LSPRs of colloidal Au NBPs can be tuned over a range of >450 nm in the extinction spectrum. These findings provide a simple and efficient approach for tailoring the innovative Au NBP synthesis and open up new opportunities for developing novel applications, such as photocatalysts for nitrogen fixation or photothermal therapy.

## Supporting Information Available

This section provides more information and the experiment results related to this study, which are not listed in the manuscript. Low magnification image, calculated electric field distributions, effect of citric acid, TEM images, lengths and width size distribution, and IR thermal images (PDF)
