# Supplementary material for: Optimization of the Surfactant Ratio in the Formation of Penta-Twinned Seeds for Precision Synthesis of Gold Nanobipyramids with Tunable Plasmon Resonances
Source: J Phys Chem C Nanomater Interfaces. 2025 Feb 14;129(8):4303–12. doi: 10.1021/acs.jpcc.4c08818 (PMC11873936; doi:10.1021/acs.jpcc.4c08818)
Supplement: Supplementary file 1 — jp4c08818_si_001.pdf [file jp4c08818_si_001.pdf]

Supporting Information

Optimization of Surfactant Ratio in Formation of Penta-twinned Seeds for Precision Synthesis of Gold Nanobipyramids with Tunable Plasmon Resonances

Au Lac Nguyen,[†] *Quinn J. Griffin,*[†] *Ankai Wang,*[§] *Shengli Zou,*[§] *Hao Jing*[†, *]

[†] Department of Chemistry and Biochemistry, George Mason University, Fairfax, Virginia 22030, USA

[§] Department of Chemistry, University of Central Florida, Orlando, Florida 32816, USA

* To whom correspondence should be addressed.
Email: hjing2@gmu.edu; Phone: 1-703-993-5221; Fax: 1-703-993-1040.

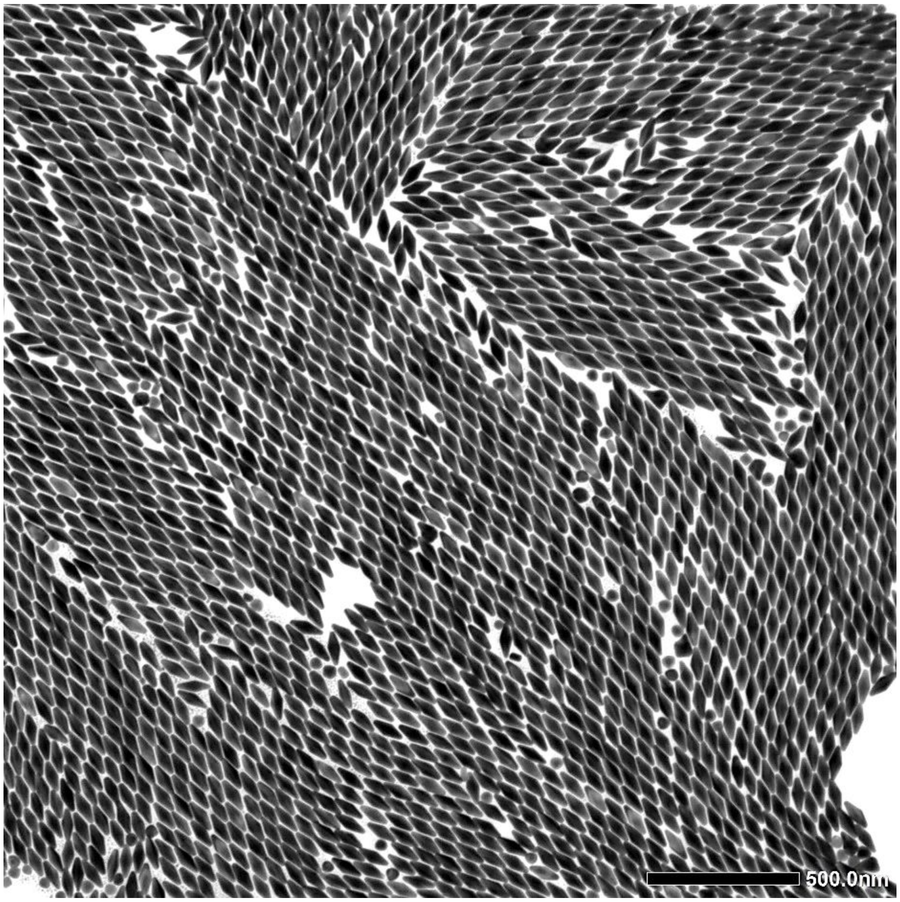

Figure S1. Low magnification image of gold nano bipyramid sample synthesized using gold seed solution with CTAC: $CiNa_3$ ratio of 21:1

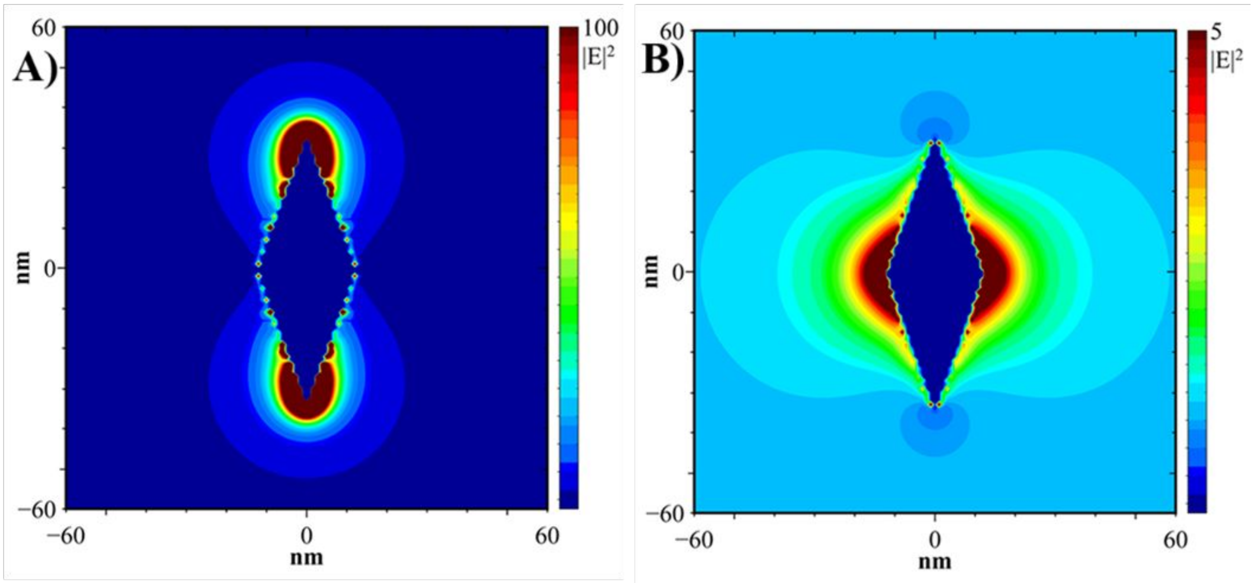

Figure S2. The calculated electric field distributions of each LSPR peak. A) The longitudinal plasmon is excited, B) the transverse one is excited

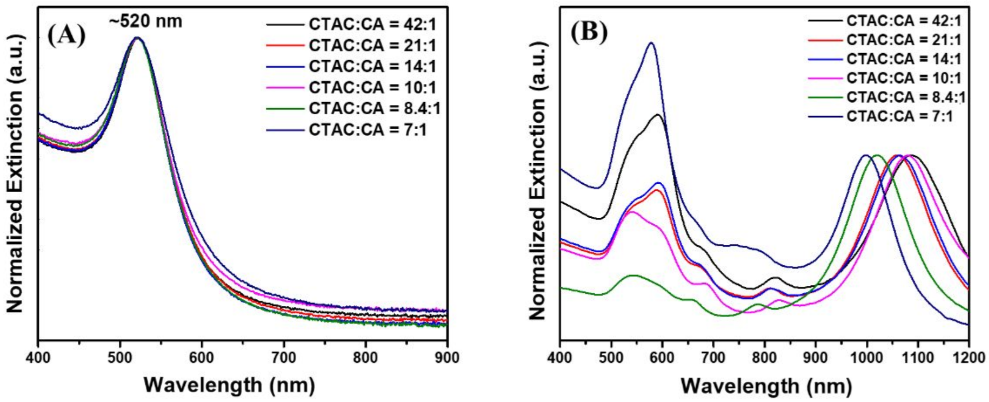

Figure S3. Effect of citric acid (CA) on the quality of gold nano bipyramids. A) UV- Vis spectra of seed solutions prepared using CA instead of $CiNa_3$ with the same molar ratio to CTAC, b) UV-Vis-NIR spectra of samples prepared using seed solution series with varying CTAC:CA ratios.

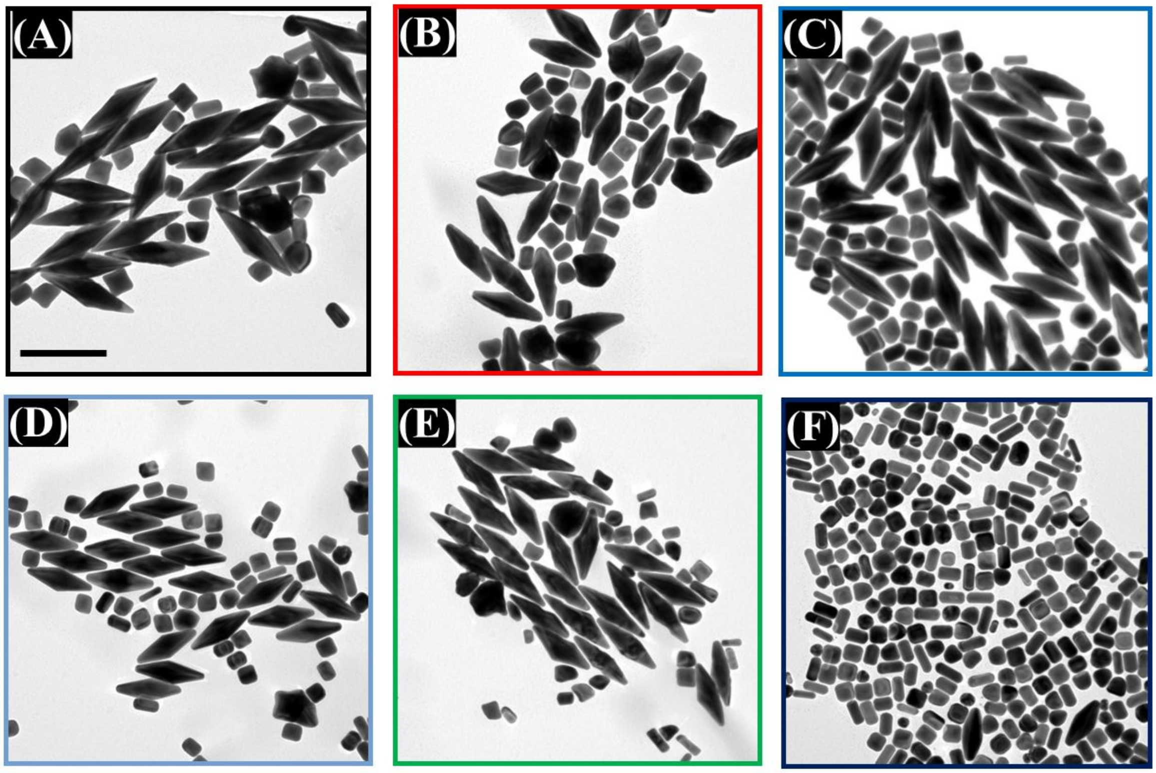

Figure S4. TEM images of monodisperse gold bipyramids synthesized at various CTAC: CA molar ratios. (A) 42:1; (B) 21:1; (C) 14:1; (D) 10:1; (E) 8.4:1; (F) 7:1. The Scale bar 200 nm

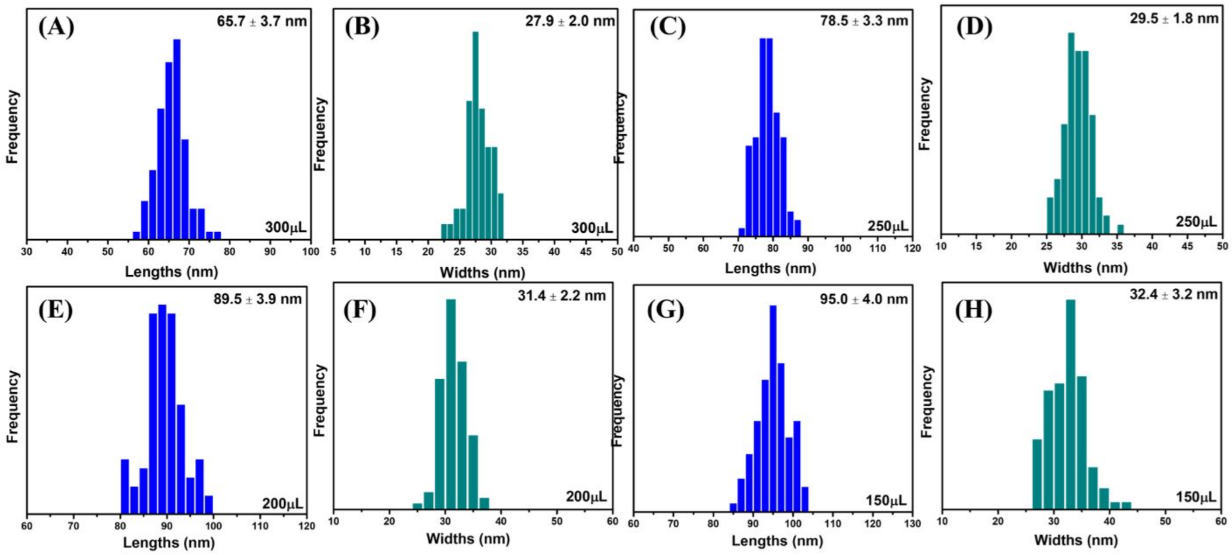

Figure S5. Lengths and width size distribution histograms of Au NBPs synthesized using varying amounts volume of optimized seed solution. A), B): 300µL; C), D): 250µL; E), F): 200µL; G), H): 150µL seed solution. Measured from around 100 particles in each sample.

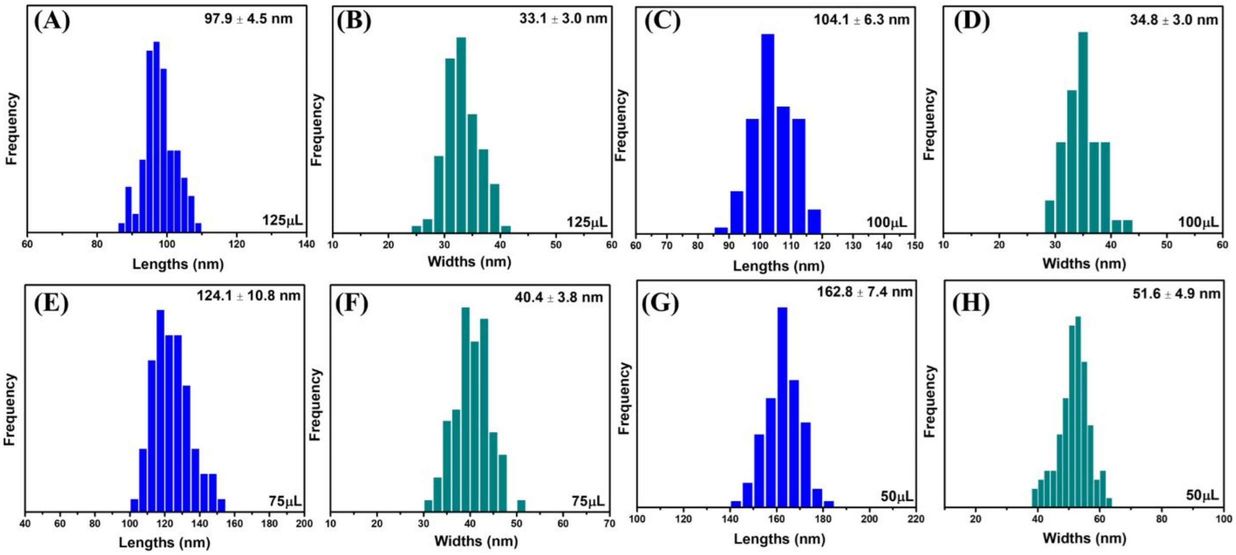

Figure S6. Lengths and width size distribution histograms of Au NBPs synthesized using varying amounts volume of optimized seed solution. A), B): 125µL; C), D): 100µL; E), F): 75µL; G), H): 50µL seed solution. Measured from around 100 particles in each sample.

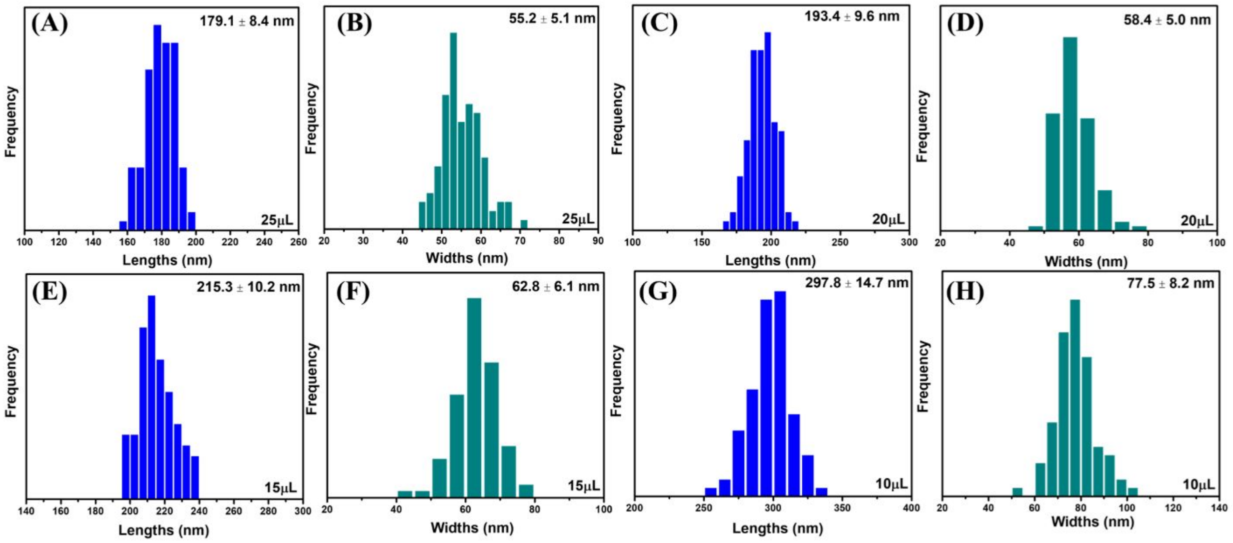

Figure S7. Lengths and width size distribution histograms of Au NBPs synthesized using varying amounts volume of optimized seed solution. A), B): 25μL; C), D): 20μL; E), F): 15μL; G), H): 10μL seed solution. Measured from around 100 particles in each sample.

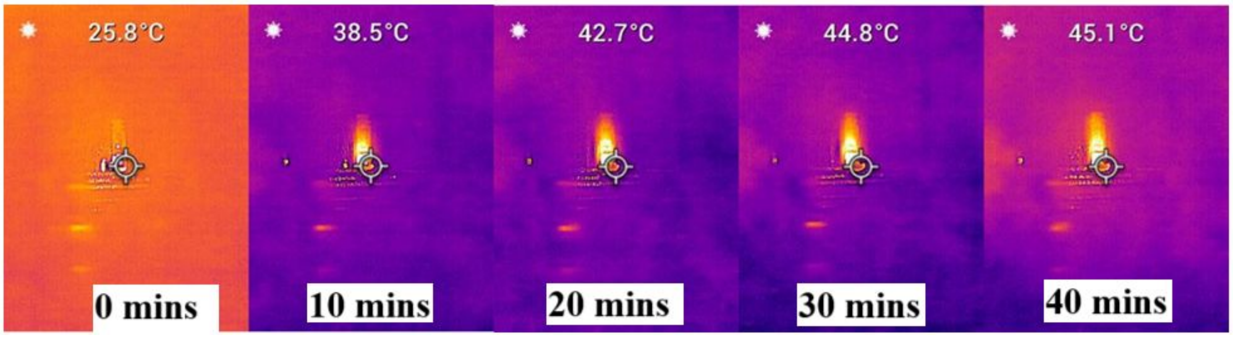

Figure S8. The IR thermal images of Au NBPs sample by irradiating with an 808nm laser were captured by an infrared (IR) thermal imaging camera (TG165-X, FLIR, Taiwan) every 10 minutes. The temperature reached its peak after 40 minutes of irradiation by an 808 nm laser.
